# Heterogeneous *EGFR* Gene Copy Number Increase Is Common in Colorectal Cancer and Defines Response to Anti-EGFR Therapy

**DOI:** 10.1371/journal.pone.0099590

**Published:** 2014-06-18

**Authors:** Annika Ålgars, Tuulia Avoranta, Pia Österlund, Minnamaija Lintunen, Jari Sundström, Terhi Jokilehto, Ari Ristimäki, Raija Ristamäki, Olli Carpén

**Affiliations:** 1 Department of Oncology and Radiotherapy, Turku University Hospital, Turku, Finland; 2 Department of Oncology, Helsinki University Central Hospital, Helsinki, Finland; 3 Department of Oncology, University of Helsinki, Helsinki, Finland; 4 Department of Pathology, University of Turku, Turku, Finland; 5 Department of Pathology, Turku University Hospital –Tyks-Sapa, Turku, Finland; 6 Division of Pathology and Genetics, Helsinki University Central Hospital - HUSLAB, Helsinki, Finland; 7 Department of Pathology, Haartman Institute and Genome-Scale Biology, Research Programs Unit, University of Helsinki, Helsinki, Finland; The Chinese University of Hong Kong, Hong Kong

## Abstract

Anti-EGFR therapy is commonly used to treat colorectal cancer (CRC), although only a subset of patients benefit from the treatment. While *KRAS* mutation predicts non-responsiveness, positive predictive markers are not in clinical practice. We previously showed that immunohistochemistry (IHC)-guided *EGFR* gene copy number (GCN) analysis may identify CRC patients benefiting from anti-EGFR treatment. Here we tested the predictive value of such analysis in chemorefractory metastatic CRC, elucidated *EGFR* GCN heterogeneity within the tumors, and evaluated the association between *EGFR* GCN, *KRAS* status, and anti-EGFR antibody response in CRC cell lines. The chemorefractory patient cohort consisted of 54 *KRAS* wild-type (WT) metastatic CRC patients. *EGFR* GCN status was analyzed by silver *in situ* hybridization using a cut-off value of 4.0 *EGFR* gene copies/cell. *KRAS*-WT and *KRAS* mutant CRC cell lines with different *EGFR* GCN were used in *in vitro* studies. The chemorefractory CRC tumors with *EGFR* GCN increase (≥4.0) responded better to anti-EGFR therapy than *EGFR* GCN (<4.0) tumors (clinical benefit, *P* = 0.0004; PFS, HR = 0.23, 95% CI 0.12–0.46). *EGFR* GCN counted using EGFR IHC guidance was significantly higher than the value from randomly selected areas verifying intratumoral *EGFR* GCN heterogeneity. In CRC cell lines, *EGFR* GCN correlated with EGFR expression. Best anti-EGFR response was seen with *KRAS*-WT, *EGFR* GCN = 4 cells and poorest response with *KRAS*-WT, *EGFR* GCN = 2 cells. Anti-EGFR response was associated with AKT and ERK1/2 phosphorylation, which was effectively inhibited only in cells with *KRAS*-WT and increased *EGFR* GCN. In conclusion, IHC-guided *EGFR* GCN is a promising predictor of anti-EGFR treatment efficacy in chemorefractory CRC.

## Introduction

Epidermal growth factor receptor (EGFR) signaling is commonly activated in colorectal cancer (CRC). EGFR-targeting monoclonal antibodies (mAb) have become a standard treatment option, particularly in the chemorefractory phase of metastatic disease [1]. *KRAS,* a signaling molecule downstream of EGFR, is mutated in approximately 40% of CRCs [1] and these activating mutations convey anti-EGFR treatment resistance [2]. In *KRAS* wild-type (WT) tumors, objective response is achieved in every third patient indicating that other factors contribute to drug efficacy [3]. Thus, there is urgent need for novel predictive markers.

*EGFR* gene copy number (GCN) increase has been linked to anti-EGFR treatment response. Most studies have shown an association between GCN level and clinical benefit, progression free survival (PFS), and in some cases, with overall survival (OS) [4]–[6]. However, *EGFR* GCN is not currently utilized in the clinical context because of technical obstacles and considerable variation between the scoring systems [7]. We recently reported a novel algorithm, which may improve the predictive value of *EGFR* GCN. We first showed that the *EGFR* GCN as analyzed by silver *in situ* hybridization (SISH) positively correlated with immunohistochemistry (IHC), when the evaluation was performed from tumor areas of highest staining intensity [8]. We further demonstrated that an increased *EGFR* GCN, using cut-off value (≥4.0), correlated positively with response to anti-EGFR therapy in all three parameters analyzed: clinical benefit, PFS, and OS. *EGFR* GCN was independent of *KRAS* status, and when the two analyses were combined, they predicted treatment response better than either test alone. The mean *EGFR* GCN, as analyzed by this method was 5.5, and copy number increase ≥4.0 was seen in 64% of the tumors. The GCN increase was typically associated with Chromosome 7 polysomy, whereas high level amplification was rarely seen.

A reason for the variation in published *EGFR* GCN results may be tumor heterogeneity, which has not been addressed in earlier studies. There is some evidence that EGFR may be heterogeneously expressed within individual colorectal tumors, both at gene and protein level [5]. Heterogeneity may complicate the analysis of EGFR protein expression and copy number alterations and lead to poor test reproducibility [7], [9]. This may be especially relevant in FISH-based analysis, where the GCN counting cannot be correlated with histology [7]. If *EGFR* heterogeneity plays a biological role, then an algorithm, in which protein expression (IHC) guides *EGFR* GCN evaluation, could improve the predictive value.

The aim of this study was to test the novel *EGFR* GCN method in an independent patient cohort and to assess the impact of *EGFR* GCN status with outcome in a combined chemorefractory patient cohort. In both patient cohorts an *EGFR* increase (≥4.0) was shown to associate with an improved clinical outcome, including clinical benefit rate, PFS, and OS. Secondary aims were to elucidate *EGFR* GCN heterogeneity within the tumors and to test, whether CRC cell lines with various *EGFR* GCN, respond differently to EGFR mAbs. According to our results, *EGFR* GCN is heterogeneous in CRC and the values obtained with IHC guidance from selected tumor areas are higher than the ones obtained by random selection. Importantly only the *EGFR* GCN counted with EGFR IHC guidance was able to predict response to anti-EGFR treatment. Our *in vitro* studies support our clinical findings, since the best response to anti-EGFR treatment was seen in *KRAS* WT, *EGFR* GCN = 4.0 cells and poorest response in the *KRAS* WT, *EGFR* GCN = 2 cells.

## Materials and Methods

### Patients

The original Turku University Hospital discovery cohort has been reported [8]. The validation cohort consisted of 31 *KRAS*-WT patients treated with EGFR mAbs in second to sixth line at the Helsinki University Hospital for metastatic CRC between June 2008 and July 2010. The selection criteria for this study were: (I) tissue was available from the primary tumor at diagnosis prior to start of any treatment, (II) the tumor was *KRAS*-WT, (III) the patients received second- to sixth-line treatment with cetuximab or panitumumab with or without chemotherapy, and (IV) the patients had no other malignancy in their history. The histology of each validation cohort case was re-evaluated by an expert in GI-pathology (AR).

The combined cohort of chemorefractory patients included 54 patients, 25 from the original Turku cohort and 29 from the Helsinki validation set. The chemorefractory patient cohort included patients treated with anti-EGFR mAbs in third line or more, either as single therapy or in combination with irinotecan +/− a fluoropyrimidine. Key characteristics of all three cohorts are described in Table 1.

**Table 1:** Characteristics of anti-EGFR treated *KRAS* wild type metastatic colorectal cancer patients.

	(a) Original discovery patient cohort ( = 44)	(b) Independent validation patient cohort (*n* = 31)	(c) Combined chemorefractory patient cohort (*n* = 54)
Median age in years (range)	60 (34–73)	63 (37–81)	61 (37–81)
		***n*** ** (%)**	
Turku University Hospital	44 (100)	-	25 (46.3)
Helsinki University Hospital	-	31 (100)	29 (53.7)
**Sex**			
Female	18 (40.9)	14 (45.2)	21 (38.9)
Male	26 (59.1)	17 (54.8)	33 (61.1)
**Site of primary tumor**			
Colon	32 (72.7)	21 (67.7)	38 (70.4)
Rectum	12 (27.3)	10 (32.3)	16 (29.6)
**Tumor differentiation grade**			
Grade 1	6 (13.6)	4 (12.9)	7 (13.0)
Grade 2	28 (63.7)	21 (67.7)	36 (66.7)
Grade 3	6 (13.6)	4 (12.9)	6 (11.1)
Unknown	4 (9.1)	2 (6.4)	5 (9.2)
**Stage of disease at diagnosis**			
Stage I	-	1 (3.2)	1 (1.8)
Stage II	9 (20.4)	2 (6.4)	9 (16.7)
Stage III	11 (25.0)	9 (29.0)	13 (24.1)
Stage IV	24 (54.6)	19 (61.3)	31 (57.4)
**Anti-EGFR therapy**			
Cetuximab	35 (79.5)	13 (41.9)	31 (57.4)
Panitumumab	8 (18.2)	16 (51.6)	21 (38.9)
Both	1 (2.3)	2 (6.5)	2 (3.7)
**Line of therapy**			
First	5 (11.4)	-	-
Second	12 (27.3)	2 (6.4)	-
Third or more	27 (61.3)	29 (93.5)	54 (100)
**Anti-EGFR combination therapy**			
Anti-EGFR combined to IRI	32 (72.7)	23 (74.2)	43 (79.6)
Anti-EGFR combined to CAP	1 (2.3)	-	-
Anti-EGFR combined to OXA	8 (18.2)	-	-
Single treatment	3 (6.8)	8 (25.8)	11 (20.4)

CAP, capecitabine; EGFR = epidermal growth factor receptor; IRI, irinotecan; OXA, oxaliplatin.

Original discovery patient cohort (**a**). Independent validation patient cohort (**b**). Combined chemorefractory patient cohort (**c**).

The response to anti-EGFR treatment was evaluated by computed tomography (CT) or magnetic resonance imaging (MRI) according to the Response Evaluation Criteria in Solid Tumors (RECIST version 1.1) [10]. Clinical benefit was considered partial response (PR) or at least 3 months of stable disease (SD). Progression free survival (PFS) was calculated from the onset of anti-EGFR treatment until disease progression. Overall survival (OS) was calculated from the onset of anti-EGFR therapy until death of any cause.

### Ethics Statement

The study was conducted in accordance with the Declaration of Helsinki. The clinical data were retrieved and histological samples collected and analyzed with the endorsement of the National Authority for Medico-Legal Affairs as well as the Institutional Review Board of the Hospital District of Southwest Finland and Ethical Review Board at Helsinki University Hospital. Written or oral informed consent was not obtained due to the fact that a large portion of the patients included in this retrospective study had died of their disease. The need for informed consent from participants was waived by the National Authority for Medico-Legal Affairs.

### IHC and SISH Procedures

*KRAS* mutation analysis was performed with the DxS KRAS mutation kit (DxS Ltd, Manchester, UK). Detailed methods for EGFR IHC and *EGFR* GCN have been described [8]. In brief, three µm sections were first stained with EGFR (clone 5B7) mAb (Ventana Medical Systems/Roche Diagnostics, Tucson AZ, USA). Stainings were performed with BenchMark XT (Ventana/Roche) using *ultra*VIEW Universal DAB Detection Kit (Ventana/Roche). *EGFR* gene was detected from subsequent five µm sections with *EGFR* DNA Probe (Ventana/Roche) and *ultra*VIEW SISH Detection Kit (Ventana/Roche). In each tumor, *EGFR* GCN of forty tumor cells was analyzed using a 40x objective by two observers (ML, JS) from areas of highest IHC reactivity. The investigators were blinded of the clinical information. To evaluate the average *EGFR* GCN within each tumor, five tumor areas were arbitrarily chosen. From each of these areas, *EGFR* GCN of 20 randomly selected cancer cells was counted by two observers (TA, JS). The results were reported as mean and range within each area.

### Cell Lines, Western Blotting and Cytotoxicity Assays

The C2BBe1, SK-CO-1 and NCI-H747 cell lines were purchased from ATCC (Manassas, VA, USA) and the CW-2 cell line from RIKEN bioresource center (Tsukuba, Japan). The NCI-H747 and CW-2 cells were cultured in RPMI-1640, the SK-CO-1 cells in EMEM and C2BBe1 cells in DMEM supplemented with 0.01 mg/ml human transferrin (Sigma, Saint Louis, MO, USA). All media were supplemented with 10% FBS, 2 mM glutamine and 1% penicillin/streptomycin.

For the signaling pathway analysis cells were grown on 6-well-plates and allowed to attach for 12 hours in normal medium. Then they were changed into medium with 1% FBS and given 0–200 µg/ml cetuximab (Erbitux, Merck Serono) for 24 hours. 25 mg/ml EGF was given to the cells for five minutes before lysis.

Protein levels were analyzed by Western blotting of cells lysed in RIPA buffer (50 mM Tris-HCl, 150 mM NaCl, 1% Nonidet P-40, 0.5% deoxycholate, 0.1% SDS, 1 mM orthovanadate, and protease inhibitor mixture, pH 7.4). Proteins were resolved by SDS-PAGE and transferred to nitrocellulose membranes. After overnight incubation in +4C° with primary antibody [anti-EGFR (D38B1, Cell Signaling Technology, Danvers, MA, USA), phospho-EGFR (Tyr1173, 53A5, Cell Signaling Technology), ERK2 (K-23, Santa Cruz Biotechnology, Dallas, TX, USA), phospho- ERK1/2 (Thr202/Tyr204, D13.14.4E, Cell signaling Technology), phospho-AKT (Ser 473), panAKT (C67E7, Cell Signaling Technology)], the membranes were incubated for 1 hour at room temperature with horseradish peroxidase-conjugated secondary antibody (Dako, Glostrup, Denmark) and the signals were detected with SuperSignal West Pico chemiluminescent substrate (Thermo Scientific, Waltham, MA, USA). Anti-α-tubulin mAb (B-1–5-1–2, Sigma) or HRP-conjugated anti-GAPDH mAb (mAbcam 9484, Abcam, Cambridge, UK) was used as a loading control.

For cell viability assays, 5000 cells/well were plated on 96-well plates. After overnight incubation in the presence of 2% FBS, the cells were exposed to 0–200 µg/ml cetuximab (Erbitux, Merck Serono) or panitumumab (Vectibix, Amgen) for 72 hours in medium supplemented with 1% FBS. Each treatment was done in triplicate and the experiment was repeated four times. Cell viability was assessed with the CellTiter 96 Aqueous One Solution Cell Proliferation assay (Promega, Madison, WI, USA). The cell viability is given as percentage of the control cells.

### Statistical Analysis

Statistical analyses were performed with the SAS 9.2 and Enterprise Guide 4.2 programs (SAS Institute Inc., Cary, NC). Frequency table data were analyzed with the χ2-test or Fisher’s exact test. The difference in *EGFR* GCN values obtained by different evaluation methods (normally distributed variables) was calculated with the Students t-test. Kaplan-Meier and log-rank tests as well as Cox proportional hazards regression model were used for univariate survival analysis. All statistical tests were two-sided. *P*-values<0.05 were considered to be statistically significant. The statistical significance for the cell viability assays was calculated with Microsoft Excel 2011 and StatPlus:mac LE (Version 2009, AnalystSoft Inc.). The significance between the differences in the responses in the cell lines was determined with two-way ANOVA followed by multiple t-tests.

## Results

### *EGFR* GCN Test Validation with an Independent Patient Cohort

**Figure 1:**
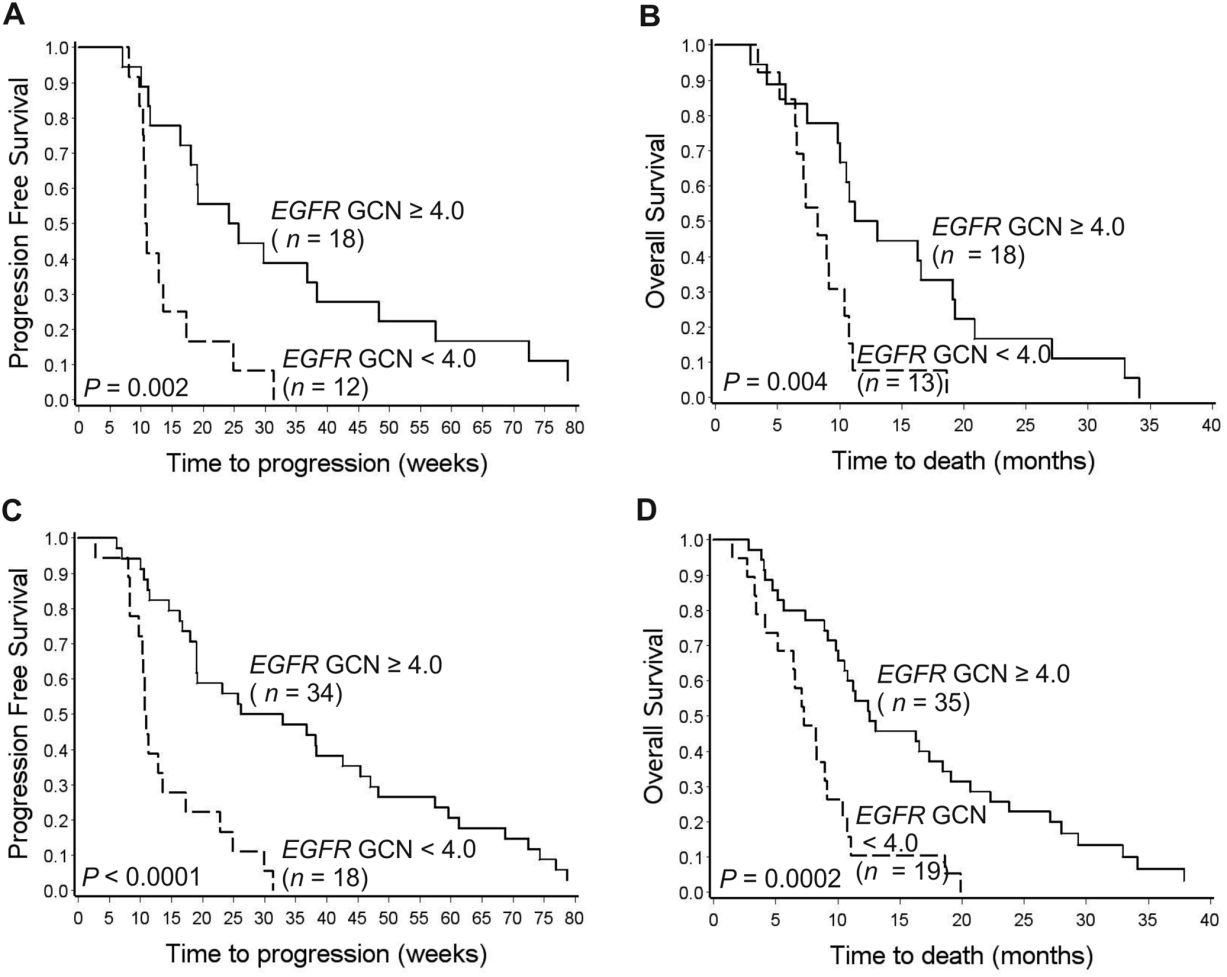
Kaplan Meier survival curves of *KRAS* wt colorectal cancer patients treated with anti-EGFR therapy. Progression free survival (**a**) and overall survival (**b**) of the test validation cohort according to *EGFR* gene copy number. Progression free survival (**c**) and overall survival (**d**) of the combined chemorefractory patient cohort.

To validate our earlier results on the association between *EGFR* GCN and anti-EGFR treatment response, we studied an independent patient cohort treated at the Helsinki University Hospital. The methods for *EGFR* GCN detection and the cut-off value for GCN increase were identical to the discovery study [8]. In the validation cohort, 18 out of 31 (58%) tumors had an *EGFR* GCN above the cut-off value (≥4.0), as compared to 64% in the original discovery set. Fourteen (78%) of the high *EGFR* GCN (≥4.0) *KRAS-*WT patients showed clinical benefit [partial response (PR) + stable disease (SD)] from anti-EGFR therapy, whereas only 4 (31%) of low *EGFR* GCN (<4.0) benefited from treatment (Chi-square Test, *P* = 0.009). An elevated *EGFR* GCN associated significantly with improved survival. The median PFS time of *EGFR* GCN≥4.0 was 25 weeks compared to only 11 weeks of the *EGFR* GCN<4.0 patients (Log-Rank test, *P* = 0.002; Cox test, *P* = 0.003, HR = 0.28, 95% CI 0.12–0.65; Figure 1a). Furthermore, the *EGFR* GCN≥4.0 associated significantly with improved OS (median 12.1 versus 8.2 months; Log-Rank test, *P* = 0.004; Cox test, *P* = 0.006, HR = 0.32, 95% CI 0.14–0.72; Figure 1b).

### Correlation between *EGFR* GCN and Treatment Response in Chemorefractory Patients

We combined the chemorefractory *KRAS* WT patients from both cohorts (*n* = 54) for further statistical analyses. Eighty per cent (28 out of 35) of the patients with a high *EGFR* GCN (≥4.0) achieved clinical benefit. In contrast, the clinical benefit rate was only 32% (6 out of 19) for the patients with a low *EGFR* GCN (<4.0) (Chi-Square test, *P* = 0.0004).

We analyzed separately the 31 chemorefractory patients treated with cetuximab (57%) and 21 with panitumumab (39%). Two patients were treated with both anti-EGFR antibodies sequentially and therefore excluded from the analysis. The clinical benefit rate in the patients treated with cetuximab +/− cytotoxic therapy with a high *EGFR* GCN in their primary tumors was 86% (18/21) as compared to 20% (2/10) in the group of patients with an *EGFR* GCN<4.0 (Fisheŕs Exact test, *P* = 0.0007). In the patient group treated with panitumumab +/− cytotoxic therapy clinical benefit was 67% (8/12) in the patients with a high *EGFR* GCN and 44% (4/9) in those with a low *EGFR* GCN. The results are presented more in detail in Table S1.

### Increased *EGFR* GCN Associates with Improved PFS and OS in Chemorefractory Disease

The median PFS of the chemorefractory patient cohort was significantly longer in the *EGFR* GCN≥4.0 patients than in the *EGFR* GCN<4.0 patients; 29.5 *vs.* 10.8 weeks (Log-Rank test, *P*<0.0001; Cox test, *P*<0.0001, HR = 0.23, 95% CI 0.12–0.46). The median OS time for patients with *EGFR* GCN≥4.0 tumors was 12.5 months compared to 7.2 months for those with *EGFR* GCN below the cut-off value (Log-Rank test, *P* = 0.0002; Cox test, *P* = 0.0003, HR = 0.32, 95% CI 0.17–0.59). Kaplan-Meier survival curves are shown in Figure 1 c–d.

PFS remained longer in the patients with a high *EGFR* GCN regardless of which anti-EGFR mAb was used. In the patients treated with cetuximab +/− cytotoxic therapy the median OS time was statistically significantly longer in the cohort of patients with an *EGFR* GCN≥4.0 as compared to those with an *EGFR* GCN below 4.0 (12.5 *vs.* 4.6 months; Log-Rank test *P* = 0.0006; Cox test *P* = 0.001, HR 0.25, 95% CI 0.10–0.58), whereas no statistically significant OS difference was seen in the patients treated with panitumumab. The results are shown in Table S1.

**Figure 2:**
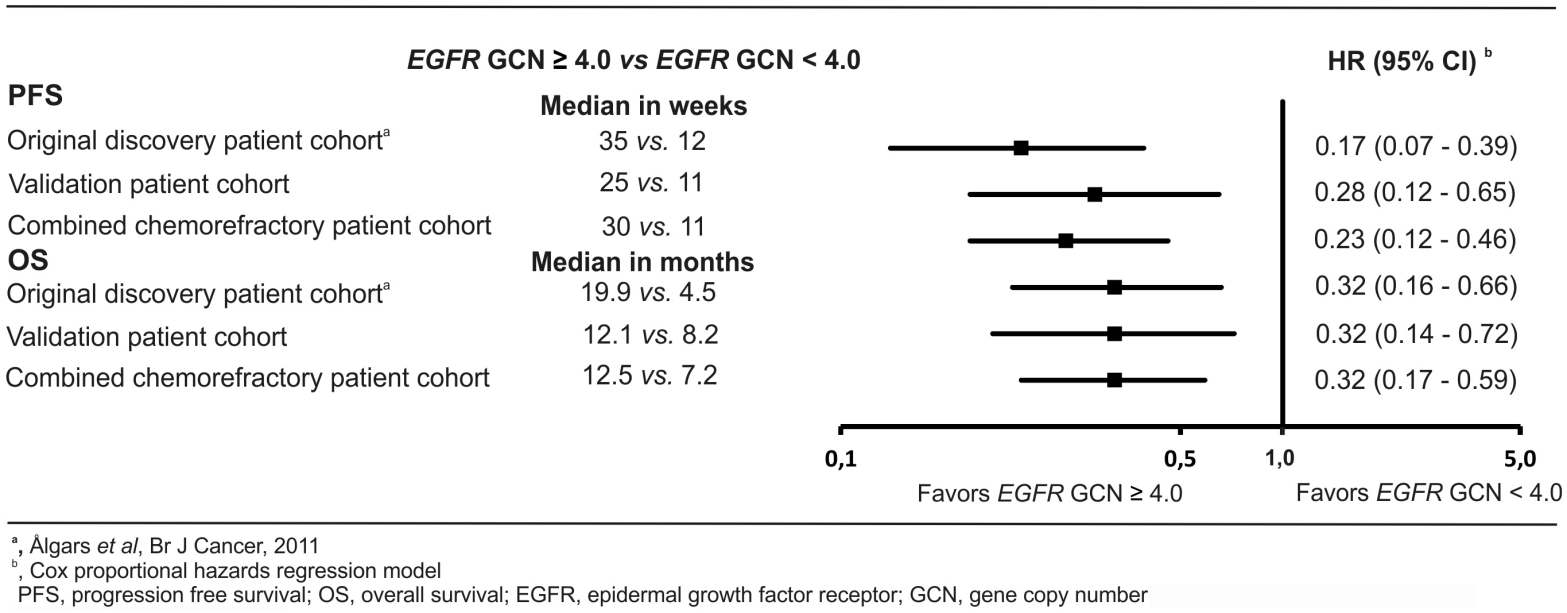
Progression-free survival and overall survival of anti-EGFR treated patients according to EGFR gene copy number. The hazard ratios and confidence intervals of the original discovery, validation, and combined chemorefractory patient cohorts are shown. A high *EGFR* GCN (IHC guided SISH) is associated with an improved disease outcome in all three *KRAS* wild type metastatic colorectal cancer patient cohorts treated with anti-EGFR therapy (two independent cohorts and one combined cohort of chemorefractory patients).

The survival data of the original discovery-, the independent validation-, and combined chemorefractory patient cohorts are compared in Figure 2.

### *EGFR* GCN Heterogeneity within Tumors

**Figure 3:**
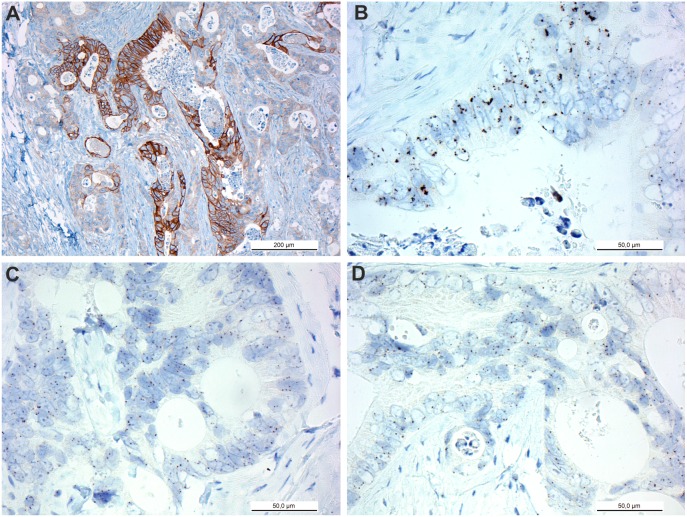
EGFR immunohistochemistry and *EGFR* silver in situ hybridization analysis in colorectal cancer. EGFR IHC shows heterogeneous staining with intensive membranous reactivity in the middle (**a)**. *EGFR* SISH from the intensively stained area showing gene clusters (**b**). *EGFR* SISH from the surrounding areas with weak or negative EGFR IHC staining shows marginally elevated or normal gene copy numbers (**c–d**).

*EGFR* GCN was randomly evaluated, without IHC guidance, from all 31 validation cohort tumors. The mean *EGFR* GCN values from randomly selected areas were significantly lower when compared to the method where the *EGFR* GCN was evaluated selectively from areas with highest EGFR protein expression (*P*<0.0001). The median *EGFR* GCN of these 31 primary CRC tumors was 4.3 when IHC guidance was used and 3.3 when the analysis was performed in a random fashion. The heterogeneity of *EGFR* GCN within one CRC tumor sample is demonstrated in Figure 3.

When the randomly chosen *EGFR* GCN values were used for survival analyses (*EGFR* GCN≥4.0 *vs. EGFR* GCN<4.0) no statistically significant difference was observed between the two groups (PFS: *P* = 0.07, HR 0.40, 95% CI 0.15–1.08; OS: *P* = 0.22, HR = 0.58, 95% CI 0.25–1.38). No significant difference in anti-EGFR treatment efficacy (clinical benefit *vs.* progressive disease) were noted either (Fisheŕs Exact test, *P* = 0.19).

### *EGFR* GCN and Response to Anti-EGFR Abs In vitro

**Figure 4:**
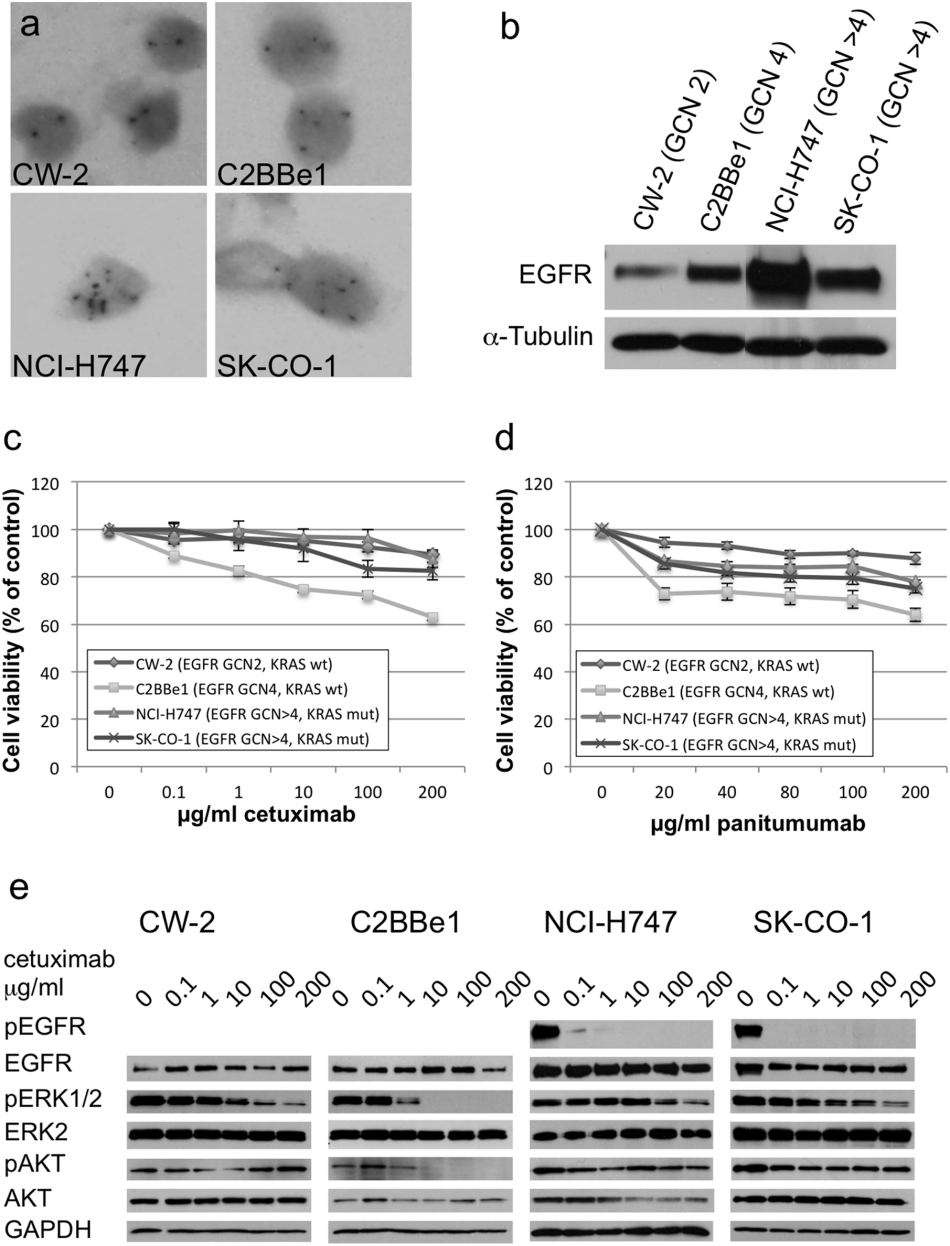
Anti-EGFR response of colorectal cancer lines with different *EGFR* GCN and *KRAS* status. (**a**) *EGFR* GCN SISH analysis of the different cell lines. (**b**) A western blot image showing the levels of EGFR protein in the different cell lines. α-tubulin was used as a control for equal loading. The cell viability of the different cell lines at varying concentrations of (**c**) cetuximab and (**d**) panitumumab. The results are given as percentage of viable cells in comparison to the non-treated cells (mean ± SE of five experiments). **(e)** Western blots showing EGFR pathway signaling molecules in the different cell lines. The cells were pretreated with the indicated amounts of cetuximab for 24 hours in medium containing 1% FBS and given egf (25 µg/ml) for 5 minutes before lysis. The indicated signaling molecules were analyzed with western blotting. GAPDH was used as a control for equal loading.

We searched the Sanger Center cancer cell line database (http://www.sanger.ac.uk/cgi-bin/genetics/CGP/cghviewer/CghHome.cgi) for *EGFR* GCN alterations. A low level of *EGFR* GCN increase (4–15 copies/cell), typically due to Chromosome 7 polysomy, was seen in 18 out of 39 (46%) cell lines. To correlate the *EGFR* GCN and *KRAS* status with anti-EGFR mAb response, we chose four lines for analysis. The CW-2 and C2BBe1 cell lines are *KRAS*-WT and have two and four copies of *EGFR,* respectively. NCI-H747 and SK-CO-1 are both *KRAS* mutant and have more than four copies (6–7) of *EGFR*. The *EGFR* GCN was confirmed by SISH (Figure 4a). The *EGFR* GCN was directly reflected in EGFR protein expression as indicated by Western blot analysis (Figure 4b). In cell viability assays, the *KRAS*-WT cell line with *EGFR* GCN 4 (C2BBe1) was the most sensitive to both cetuximab and panitumumab treatment (Figure 4c). The difference was highly significant when compared to any of the other cell lines (Student’s t-test, *P*<0.001). Interestingly, the *EGFR* disomic, WT *KRAS* cell line (CW-2) was most resistant to mAb treatment. The cell lines with mutant *KRAS* and *EGFR* GCN>4 showed intermediate sensitivity, especially to panitumumab treatment. The results are thus in agreement with our clinical data indicating that both *EGFR* GCN and *KRAS* status define the tumor cell response to anti-EGFR mAbs.

We further looked at the effect of anti-EGFR mAb treatment on intracellular signaling in these cell lines. In *KRAS* mutant cell lines with more than four *EGFR* gene copies, phosphorylation of EGFR was evident and efficiently blocked by anti-EGFR mAb (Figure 4e). Anti-EGFR inhibition partially reduced pERK1/2 levels, while the level of pAKT was not affected. In *KRAS*-WT cell lines with 2–4 *EGFR* gene copies EGFR phosphorylation was under detection limit (not shown). However, the pathway was apparently operational as indicated by responses on downstream signaling. In the *EGFR* disomic line CW-2, anti-EGFR mAb treatment reduced pERK1/2 level, but did not affect AKT phosphorylation (Figure 4e). Only in *EGFR* polysomic and wild-type *KRAS* C2BBe1 cells, an effective blockage of both ERK1/2 and AKT signaling, was detected (Figure 4e). These results thereby suggest that both *EGFR* GCN and *KRAS* mutation status determine the effect of anti-EGFR mAb on EGFR downstream signaling and colorectal cancer cell survival.

## Discussion

In this study, we demonstrate that heterogeneous *EGFR* GCN increase is a strong predictor of anti-EGFR treatment benefit in metastatic CRC. The results extend our previous findings of a single institute patient cohort to an independent validation cohort. In addition, they demonstrate the predictive value of *EGFR* GCN for anti-EGFR therapy efficacy in chemorefractory CRC patients, the most important patient group eligible for this treatment. The results further show that intra-tumoral *EGFR* GCN heterogeneity is common in CRC. We hypothesize that this previously unaccounted *EGFR* heterogeneity is a reason for the reported poor correlation between *EGFR* GCN analysis and efficacy of anti-EGFR therapy, and suggest an improved method for predictive *EGFR* GCN testing.

The patient material in our study included patients treated with anti-EGFR therapy both in an early and chemorefractory phase of metastatic CRC therapy. To control for the confounding effects of chemotherapy sensitivity when combined with anti-EGFR mAbs on our results, the predictive value of *EGFR* GCN on anti-EGFR treatment response was evaluated separately in the subgroup of patients treated in a chemorefractory phase (third line or more). The results obtained in the chemorefractory subgroup were similar to the results of the entire patient cohort, which supports our interpretation that a high *EGFR* GCN is indeed a predictor of favorable anti-EGFR treatment response.

Both cetuximab and panitumumab in the chemorefractory patient subgroup demonstrated improved PFS in *EGFR* GCN≥4.0 patients. Overall survival and disease control rate was statistically improved for the cetuximab cohort, and numerically but not statistically in the small panitumumab cohort. The two therapeutic Abs are of different type, panitumumab being a fully human mAb, whereas cetuximab is a mouse-human chimera. *In vitro* assay demonstrated identical cytotoxicity for the tested CRC lines, but *in vivo*, other mechanisms, including antibody-dependent cytotoxicity (ADCC) may be operational. In this respect, cetuximab and panitumumab may be different and therefore, increased *EGFR* GCN could better indicate sensitivity to cetuximab treatment [11]. According to pre-clinical and clinical data certain cytotoxic therapies +/− immunomodulating agents, including e.g. the GILF (gemcitabine, irinotecan, levofolinic, and 5-fluorouracil), GILFI (gemcitabine, irinotecan, levofolinic acid, fluorouracil, aldesleukin), and GOLFIG (gemcitabine, oxaliplatin, levofolinate, 5-fluorouracil, GM-CSF, aldesleukin) regimens, have the ability to effectively up-regulate EGFR expression on colon cancer cells, thereby enhancing the sensitivity of the colon cancer cells to cetuximab-mediated ADCC [12]–[14]. In our study, panitumumab was used as single therapy more often than cetuximab, 42.9 *vs* 6.5% respectively, which at least in part can explain the difference observed in the efficacy results between the two antibodies. Both antibodies, when not administered as single treatment, were combined with irinotecan based chemotherapy regimens. In our study, oxaliplatin or single capecitabine were not combined to anti-EGFR therapy in the chemorefractory phase of the disease. Two patients were given cetuximab and panitumumab sequentially and therefore excluded from this study. Therefore, neither the combination cytotoxic drug nor the administration of both antibodies sequentially, explains the difference in the results obtained with the different anti-EGFR antibodies. Alternatively, our result may reflect a difference between treated patients. Cetuximab was more commonly used in the discovery cohort, which defined the test cut-off value, and panitumumab in the validation cohort with only chemorefractory patients. As both cohorts were retrospective, we cannot exclude differences in the patient populations, which could account for the differences in treatment results.

Tumor heterogeneity has been suggested to contribute to difficulties encountered in the validation of oncology biomarkers [15]. In the present work, the mean *EGFR* GCN values were significantly lower when analyzed in a random fashion as compared to the values obtained by choosing the cells with the highest GCN. Only the latter method was able to distinguish patients according to their anti-EGFR treatment response. This supports the view that therapeutic decision making based on scoring the dominant phenotype may be misleading [15]. Even a minor percentage of mutant alleles and gene expression profiles can be crucial for treatment response [16].

*EGFR* GCN heterogeneity is a well-established phenomenon in gliomas [17]. Although *EGFR* GCN heterogeneity in CRC may have been identified earlier, this finding has been generally disregarded, and not utilized as a parameter in diagnostic analyses. In some respects, the association between *EGFR* GCN and treatment response resembles the findings of another EGFR family member, *Her2,* in gastric cancer. *Her2* is amplified or overexpressed in 7–34% of gastric cancers [18]. Unlike in breast cancer, Her2 expression in gastric cancer shows marked intra-tumoral heterogeneity, and therefore diagnostic test interpretation differs from breast cancer [19]. The current predictive diagnostics for trastuzumab treatment in gastric cancer is based on a combination of Her2 IHC and *Her2* GCN analysis of areas with highest IHC staining. In biopsies, such areas may cover just 5% of the tumor [19]. Although there are parallels, the Her2-gastric cancer algorithm differs from *EGFR* GCN in CRC. In gastric cancer, the cut-off value is based on gene to chromosome ratio ≥2.0, although most recent recommendations suggest that *Her2* GCN>6.0 can be considered as positive. In CRC, the *EGFR* GCN increase is typically a result of chromosome 7 polysomy and therefore the gene to chromosome ratio is not informative. On the other hand chromosome 17 is rarely polyploid in gastric cancer, which indicates a different biological mechanism for *EGFR* and *Her2* increase.

Tumor heterogeneity has been suggested to underlie resistance to targeted cancer therapeutics [15]. In this respect, the finding that a minor population of *EGFR* GCN high cells defines treatment response is unexpected. As the *EGFR* GCN low cells do not respond to treatment (the *EGFR* GCN low tumors are unresponsive), the most obvious outcome would be treatment failure, when the *EGFR* GCN low cells take over. This appears, however, not to be the case, therefore alternative explanations must be considered. One possibility is that the *EGFR* GCN high cells possess biological activities that determine the outcome of the entire tumor cell population. In such case, *EGFR* high cells may have yet undefined properties, such as paracrine effects on the major tumor cell population or features of cancer stem cells. There is evidence that, indeed, the transcriptome of the *EGFR* GCN high cells differs from EGFR low cells and that the EGFR pathway is associated with intestine stem cell properties [20], [21]. At present, the mechanism by which *EGFR* GCN high cells dictate treatment response remains unknown.

EGFR signaling results in activation of intracellular pathways such as Ras/Raf/MAPK/ERK and PI3K/AKT, which regulate cell proliferation and survival. Anti-EGFR mAb:s are suggested to exert their anti-cancer effects by blocking these pathways [22]. Our cell line studies suggested a mechanism, by which the combination of *EGFR* GCN and *KRAS* mutation status can regulate the response to anti-EGFR treatment. EGFR inhibition with mAbs led to suppression of AKT pathway activity only in cells with increased *EGFR* GCN and *KRAS*-WT. Although EGFR phosphorylation was effectively blocked in *KRAS* mutant cells, AKT signaling was not suppressed, apparently due to constitutively active *KRAS*. Interestingly, in *EGFR* disomic and *KRAS*-WT cells, AKT signaling was active but not responding to anti-EGFR mAb suggesting that cells with normal *EGFR* GCN utilize alternative mechanisms to sustain AKT signaling. Similarly, the ERK1/2 phosphorylation was fully supressed only in cells with increased *EGFR* GCN and WT *KRAS*. In conclusion, this study indicates that the diagnostic algorithm combining EGFR IHC and *EGFR* SISH is a highly promising method for selecting CRC patients benefiting from anti-EGFR treatment. When combined with *KRAS* mutation testing, the ratio of responsive patients in the test positive group may almost double as compared to *KRAS* test alone. If further test validation using prospectively collected and randomized patient cohorts confirms the test results, these findings may have a profound effect on future selection of CRC patients for anti-EGFR therapy.

## Supporting Information

Table S1 Tumor response and survival of patients with chemorefractory *KRAS* wild type metastatic or locally advanced colorectal cancer treated with cetuximab or panitumumab according to *EGFR* Gene Copy Number.
(XLS)
